# Whole-genome assembly and annotation of the acorn weevil, *Curculio nanulus* (Coleoptera: Curculionidae)

**DOI:** 10.1093/g3journal/jkaf292

**Published:** 2025-12-06

**Authors:** Daniel D Davis, Michael A Charles, Duane D McKenna, Paul B Frandsen

**Affiliations:** Department of Plant and Wildlife Sciences, Brigham Young University, Provo, UT 84602, United States; Department of Biological Sciences, University of Memphis, Memphis, TN 38152, United States; Center for Biodiversity Research, University of Memphis, Memphis, TN 38152, United States; Department of Biological Sciences, University of Memphis, Memphis, TN 38152, United States; Center for Biodiversity Research, University of Memphis, Memphis, TN 38152, United States; Department of Plant and Wildlife Sciences, Brigham Young University, Provo, UT 84602, United States; Life Science Museum, Brigham Young University, Provo, UT 84602, United States

**Keywords:** *Curculio nanulus*, genome assembly, seed parasitism, comparative genomics, pacBio hiFi

## Abstract

The acorn weevil *Curculio nanulus* (Coleoptera: Curculionidae) is a seed predator that lays its eggs inside developing acorns and hickory nuts in the western United States. The female weevil uses her elongated rostrum to excavate a hole into the seed, creating a protected site for oviposition. Natural history traits among *Curculio* species—such as host specificity and variation in larval diapause—suggest a dynamic evolutionary relationship with their host plants. These traits are best studied through a comparative genomic framework, but such analyses cannot currently be undertaken due to the lack of whole-genome assemblies for *Curculio* species. To address this gap, we generated a whole-genome assembly for *C. nanulus* using PacBio HiFi sequencing. The resulting assembly is ∼1.5 Gbp in length, with high contiguity (contig N50 = 7.7 Mbp) and gene completeness (BUSCO score: 98.97%). To enable comparative analysis, we also assembled the genome of the pecan weevil, *Curculio caryae*, using publicly available PacBio HiFi reads. For both species, we annotated repetitive elements and protein-coding genes and compared these features with those of other weevil genomes. Our results reveal a marked expansion of repetitive elements within *Curculio* and its close relatives. These genomic resources provide a foundation for investigating seed predation, co-speciation, and host-parasite evolutionary dynamics in *Curculio* and related taxa, as well as their impacts on forest ecology.

## Introduction

Nut and acorn weevils of the genus *Curculio* (Coleoptera: Curculionidae) comprise a diverse lineage of seed predators with a distinctive life history. Females use their elongated rostrum to bore into developing hard-shelled seeds—typically those of oak, hazel, hickory, or chestnut—to deposit their eggs within the seed's interior (Matsumura et al. 2021). This protected, nutrient-rich environment promotes larval development, but the presence of larvae simultaneously compromises the host seed's viability, often preventing germination (Higaki 2016).

In response to such predation pressure, many oak species have evolved masting behavior—synchronized cycles of high and low seed production across populations (Kelly 1994). During mast years, trees produce a surplus of acorns, exceeding the consumption capacity of local seed predators like *Curculio*. In non-mast years, the reduced seed output curtails weevil reproduction, potentially lowering population densities (Higaki 2016). This cyclical mismatch between resource availability and consumer abundance represents an ecological and evolutionary strategy to limit seed predation.

*Curculio* species, in turn, have evolved counter-adaptations. After larvae exit their host seeds, they enter a period of diapause within the soil, which may last for one or more years. This dormancy allows populations to persist through low-resource periods and potentially synchronize emergence with future mast events. The duration and dynamics of diapause vary widely both among and within *Curculio* species (Menu and Desouhant 2002; Higaki 2016; Espelta et al. 2017), suggesting ongoing evolutionary adaptation to host reproductive cycles (Maeto and Ozaki 2003).

This co-evolutionary relationship between host and seed predator has likely shaped key traits such as host specificity, life cycle timing, and ecological resilience. Despite their ecological significance, the genetic basis of these traits in *Curculio* remains poorly understood. The lack of whole-genome assemblies for *Curculio* species has prevented investigations into the molecular underpinnings of diapause regulation, host–plant interactions, and adaptive evolution.

To address this, we sequenced and assembled the genome of *Curculio nanulus*, a western U.S. acorn weevil, using PacBio HiFi technology. To enable comparative analyses, we also assembled the genome of the pecan weevil, *Curculio caryae,* using publicly available PacBio HiFi reads generated by the United States Department of Agriculture (NCBI SRA: SRR18245025). These assemblies serve as a foundation for future evolutionary and ecological studies in *Curculio* and other seed-parasitic insects.

## Methods

### DNA extraction and sequencing

We collected a larval specimen of *Curculio nanulus* from an acorn found underneath a mossycup oak tree *Quercus macrocarpa* on the Brigham Young University campus in Provo, UT, USA, in September 2022. We extracted high molecular weight genomic DNA from the specimen using the Qiagen genomic-tip DNA extraction kit. We sheared purified DNA to 18 kbp with a Diagenode Megaruptor and used a BluePippin system (Sage Science, Beverly, MA, USA) to collect fractions containing >15 kbp fragments for library preparation. We prepared genomic DNA libraries using the SMRTbell Express Template Prep Kit 2.0 and associated protocol (PacBio, Menlo Park, CA, USA) and sequenced the library on a single PacBio Revio flow cell at the BYU DNA Sequencing Center. Additionally, we downloaded publicly available PacBio HiFi sequencing reads from the NCBI SRA for the pecan weevil, *Curculio caryae* (SRR18245025), to support comparative analyses.

### Assembly and refinement

We estimated genome size, repeat content, and heterozygosity using KMC v3.1.1 for k-mer counting (Kokot et al. 2017) and GenomeScope 2.0 (Ranallo-Benavidez et al. 2020). We assembled the PacBio HiFi reads for each species using hifiasm v0.16.1-r375 (Cheng et al. 2021). We subsequently used purge_dups v1.2.5 to remove duplicated haplotigs (Guan et al. 2020). Purge_dups uses contig similarity and depth of coverage to assess whether a contig represents haplotypic variation rather than a true duplication. Contigs identified as haplotypic duplicates were excluded from the assembly.

We identified and removed contaminant contigs using BlobTools v1.1.1 (Laetsch et al. 2017; Laetsch and Blaxter 2017) based on closest taxonomic match from a blastn v2.12.0 search against the NCBI nt database, GC proportion, and sequencing coverage.

We located, circularized, and annotated the mitochondrial genomes for each species using MitoHiFi (Uliano-Silva et al. 2023) with the maize weevil, *Sitophilus zeamais*, (NC_030764.1, 18105 bp, 37 genes) (Ojo et al. 2016) mitochondrial genome as a reference. Circular representations of the contigs were visualized with OrganellarGenomeDraw v1.3.1 (Greiner et al. 2019).

### Identification

Following whole-genome sequencing, we discovered that the sequenced larval specimen could not be identified to the species level using a morphological key because no such key exists for the *Curculio* larvae of the western United States. To aid in our identification, we collected larval specimens from English oak *Quercus robur* at the same locality as the original specimen. We elected to collect specimens from a different host species because the original host tree had been cut down. We then reared the larvae to adulthood in a moist coconut coir substrate kept between 65 and 72°F. We identified adult specimens to species using the key from (Gibson 1969) and imaged the specimens with an Olympus DP75 camera mounted on an SZX-ILLB100 microscope (Supplementary Fig. 1). We sequenced a 658 bp fragment of the 5′ end of the mitochondrial gene cytochrome C oxidase subunit I (COI) for 2 identified adult and 2 larval specimens to aid in confirming the identification of the sequenced specimen. The primers used in both PCR and sequencing of the COI fragment were LCO1490-L and HCO2198-L (Nelson et al. 2007).

To determine whether the species from which we sequenced the whole genome was the same species as those we reared, we extracted the COI gene from the mitochondrial genome assembly and aligned it with the COI fragments from the identified specimens using MUSCLE as implemented in MEGA v11.0.13 (Tamura et al. 2021). MEGA v11.0.13 was used both to calculate pairwise distances and to construct the maximum likelihood tree using default parameters.

### Quality control

For each purged and decontaminated genome assembly, we used assembly_stats.py (Trizna 2020) to measure assembly statistics including N50, L50, genome size, number of contigs, and GC content. The average sequencing coverage was calculated for each assembly by dividing the assembly size by the total read lengths of the respective sequencing runs.

We used compleasm to estimate gene completeness, by estimating the number of benchmarking universal single-copy orthologs (BUSCOs) that each genome shares with the endopterygota_odb10 ortholog set (Huang and Li 2023).

### Genome annotation

We identified repetitive regions in each genome *ab initio* and analyzed repetitive elements with Earl Grey v4.4.0 (Baril et al. 2024). We completed structural annotations on the soft-masked assemblies using the GALBA v1.0.11 pipeline (Stanke et al. 2006; Buchfink et al. 2015; Hoff and Stanke 2019; Brůna et al. 2023; Li 2023) with the merged protein annotations of 6 curculionids (*Sitophilus oryzae*: GCF_002938485.1; *Dendroctonus ponderosae*: GCF_020466585.1; *Dendroctonus valens*: GCA_024550625.1; *Euwallacea fornicatus*: GCF_040115645.1; *Anthonomus grandis grandis*: GCF_022605725.1; *Rhynchophorus ferrugineus*: GCF_030347505.1) and one brentid (*Cylas formicarius*: GCF_029955315.1) as references. Completeness of each annotation was evaluated with compleasm v0.2.5 (Huang and Li 2023) in protein mode with the endopterygota_odb10 ortholog set. Functional annotations were completed using Blast2GO v1.5.1 (Conesa and Götz 2008).

### Comparison of repetitive elements

We used Earl Grey v4.4.0 (Baril et al. 2024) to annotate the repetitive elements of 6 additional curculionid genomes and one brentid genome available from GenBank (Table 1). We arranged visualizations of the repetitive elements on a phylogeny constructed using the protein sequences from compleasm annotations. To construct the phylogeny, we aligned the amino acid sequences from each single-copy compleasm locus with MAFFT v7.526. We scored the alignments with Aliscore v02.2 and trimmed them with ALICUT V2.31. We estimated the final phylogenetic tree using 2 methods: first, by concatenating the aligned sequences and conducting a partitioned model search followed by maximum likelihood construction with IQtree v2.1.3; and second, by constructing a gene tree for each locus with IQtree v2.1.3 followed by species tree estimation with Astral III v5.7.1, using the gene trees as input. The phylogeny was rooted using *Cylas formicarius* (Brentidae: Cyladinae) and then visualized using FigTree v1.4.4 (http://tree.bio.ed.ac.uk/software/figtree/). We arranged plots showing the change in the repetitive elements over time for each species on the phylogeny.

**Table 1.** Genomes used in comparison of repetitive elements.

Family	Species	GenBank accession
Curculionidae	*Anthonomus grandis grandis*	GCA_022605725.3
	*Dendroctonus valens*	GCA_024550625.1
	*Curculio caryae*	Assembled from SRR18245025
	*Curculio nanulus*	(assembled for this study)
	*Kuschelorhynchus macadamiae*	GCA_030620095.1
	*Listronotus oregonensis*	GCA_019359885.1
	*Rhynchophorus ferrugineus*	GCA_030347505.1
	*Sitophilus oryzae*	GCA_002938485.2
Brentidae	*Cylas formicarius* (outgroup)	GCA_029955315.1

### Comparison to available Curculionidae genomes

There are 38 genome assemblies representing 24 species belonging to the weevil family Curculionidae in GenBank. We evaluated all 38 genomes for contiguity using assembly_stats.py (Trizna 2020) and completeness using compleasm with the endopterygota_odb10 ortholog set. For downstream analyses, we included the best genome for each species based on the compleasm completeness statistic. The curculionid genomes that we used are available at https://www.ncbi.nlm.nih.gov/datasets/genome/?taxon=7042.

## Results

### Species verification

The COI sequence from the *Curculio nanulus* whole-genome assembly differed by no more than 0.3% from verified *C. nanulus* specimens (Supplementary Table 1). Phylogenetic analysis also placed our sequenced specimen (GenBank: JBEWYK010000000) firmly within the *C. nanulus* clade (Supplementary Fig. 2), strongly supporting its species identity.

### Genome sequencing and assembly

Sequencing of *C. nanulus* generated 81.8 Gbp of data across 5,212,942 PacBio HiFi reads. GenomeScope 2.0 predicted a genome length of 1.09 Gbp, with 55% repeat content and 2.5% heterozygosity. The initial assembly (Table 2) totaled 1.6 Gbp across 898 contigs, with 51 × sequencing coverage, a contig L50 of 57 contigs, and a contig N50 of 7.19 Mbp. Following refinement via purge_dups and BlobTools, the assembly was reduced to 1.5 Gbp across 652 contigs, improving contiguity (N50 = 7.71 Mbp; L50 = 51 contigs) and sequencing coverage (54×). The final assembly size is ∼0.41 Gbp larger than the estimate by GenomeScope 2.0.

**Table 2.** Contiguity and completeness statistics for the *Curculio nanulus* initial, intermediate and final genome assemblies as well as completeness for gene annotations. Sequencing coverage was calculated by dividing the number of base pairs in the raw sequencing reads by the assembly size. Compleasm was run with the endopterygota_odb10 database.

	*Curculio nanulus*
**Sequencing reads (bp)**	81,847,322,991
**Reads count**	5,212,942
**Mean read length**	15,700.8
**Read N50**	15,830

	Initial assembly	Post-PurgeDups	Decontaminated with Blobtools
**Contig count**	898	654	652
**L50**	57	51	51
**N50**	7,189,678	7,709,206	7,709,206
**% GC content**	35.059	35.068	35.073
**Shortest contig (bp)**	5,225	5,225	5,225
**Mean contig (bp)**	1,785,266	2,311,745.6	2,316,901.6
**Median contig (bp)**	355,929.5	549,145.5	549,145.5
**Longest contig (bp)**	24,979,329	24,979,329	24,979,329
**Total length (bp)**	1,603,168,799	1,511,881,652	1,510,619,856
**sequencing coverage**	51x	54x	54x

BUSCOs (compleasm)			
Complete (%)	99.01	98.97	98.97
Single copy (%)	95.90	97.46	97.46
Duplicated (%)	3.11	1.51	1.51
Fragmented (%)	0.24	0.24	0.24
Missing (%)	0.75	0.80	0.80

Annotation	
Putative genes	26,420
Putative transcripts	29,745
Complete (%)	97.6
Single copy (%)	75.28
Duplicated (%)	22.32
Fragmented (%)	0.8
Missing (%)	1.6

After refinement, the *C. nanulus* assembly contained 98.97% complete BUSCOs. Complete and single-copy BUSCOs increased from 95.90 to 97.46%, while duplicated BUSCOs decreased from 3.11 to 1.51%, indicating improved assembly quality and reduced haplotypic duplication, with only a minimal (∼0.04%) decrease in total completeness.

The mitochondrial genome of *C. nanulus* was 18,823 bp in size (Supplementary Fig. 3), encoding 37 genes, the same number present in the reference and *C. caryae* (20,075 bp). Gene order was generally conserved between *C. nanulus* and *C. caryae*, with only minor differences in the positions of tRNAs relative to the reference.

The *C. nanulus* assembly is highly contiguous, and contiguity improved with post-assembly refinement using purge_dups (Guan et al. 2020). Notably, despite their close relationship, the refined *C. nanulus* assembly was ∼1.51 Gbp—approximately 634 Mbp smaller than the *C. caryae* assembly (∼2.14 Gbp; Supplementary Table 2).

Further results for the *C. caryae* assembly can be found in the Supplementary material.

### Repetitive elements and genome size

**Fig. 1.**
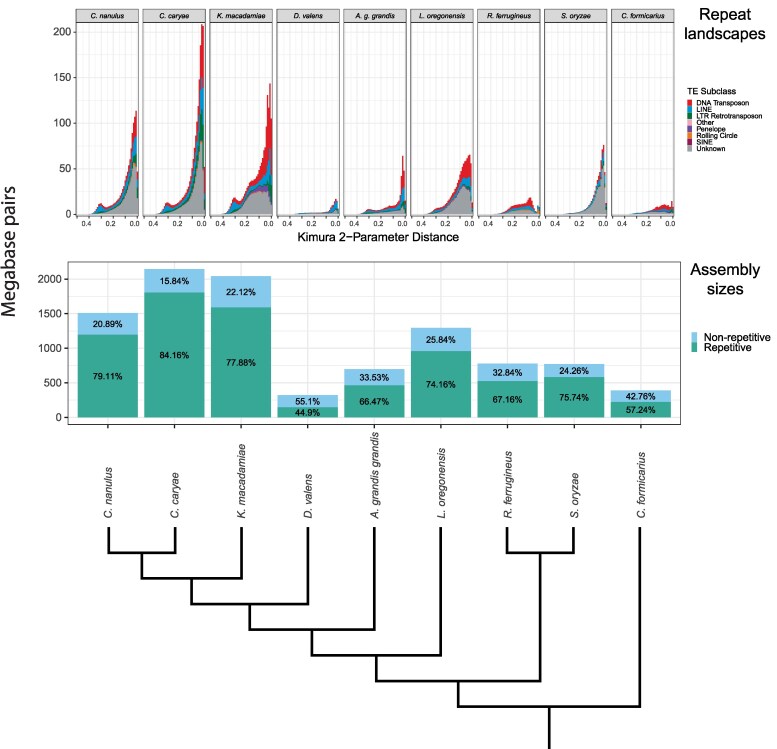
Repetitive elements identified in the genome assemblies of 8 curculionid and one brentid species. The species are ordered according to the cladogram shown. The top plot shows changes in total repetitive elements across time. The second plot compares genome assembly sizes broken into repetitive and non-repetitive DNA.

**Fig. 2.**
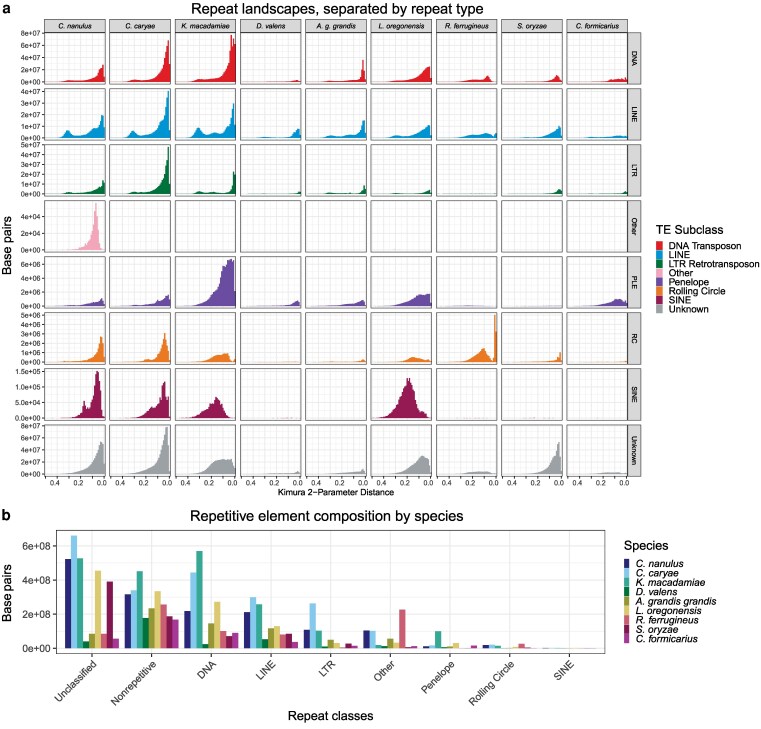
Repetitive elements of 8 curculionid and one brentid species. a) Repetitive elements across time, split into classes. b) Comparison of total base pairs of major repeat classes broken down by species. “Other” includes simple repeats, microsatellites, and repetitive RNA.

The *C. nanulus* assembly was 79.11% repetitive (1,195.1 Mbp), whereas the *C. caryae* assembly was 84.16% repetitive (1,805.1 Mbp) (Fig. 1). The sizes of non-repetitive regions were comparable: 315.5 Mbp in *C. nanulus* and 339.8 Mbp in *C. caryae* (Fig. 2b). Therefore, the 634 Mbp difference in total genome size is attributable primarily to greater expansion in the repetitive content of the *C. caryae* genome, especially unclassified repeats, DNA transposons, LINEs, and LTR retrotransposons. In both *Curculio* genomes, the majority of the repetitive elements were unclassified: 34.6% of the *C. nanulus* genome and 30.7% of the *C. caryae* genome.

The maximum likelihood tree constructed from concatenated sequences and the ASTRAL species tree had identical topologies. Mapping repeat landscapes onto this phylogeny (Fig. 1) showed a shared recent expansion of repetitive elements in the clade containing *C. nanulus*, *C. caryae*, and *Kuschelorhynchus macadamiae*. This expansion continued within *Curculio,* though certain repeat types (eg unclassified elements, SINEs, and rolling-circle elements) plateaued in *K. macadamiae* (Fig. 2a). In contrast, *K. macadamiae* exhibited substantial growth of Penelope elements, which now occupy 6 to 9 times more sequence than in *Curculio,* yet still comprise just 4.9% of the genome.

Among *Curculio*, *C. caryae* exhibited greater repetitive element expansion than *C. nanulus*, explaining its larger genome size. Except for *Dendroctonus valens*, all analyzed weevil genomes contained at least 57% repetitive content (Fig. 1). Independent repeat expansions were evident in the lineages leading to *A. grandis grandis*, *L. oregonensis*, *R. ferrugineus,* and *S. oryzae,* distinct from the *Curculio/Kuschelorhynchus* expansion.

In *C. nanulus*, unclassified elements comprised 43.7% of repetitive elements (34.6% of the genome), while in *C. caryae*, they accounted for 36.6% of repetitive elements (30.7% of the genome). This pattern aligns with findings from other weevils (eg Sylvester et al. 2024) and other non-model insects, where an average of 40.5% of repeats remain undescribed outside of *Drosophila* (Sproul et al. 2023).

### Gene annotation

The *C. nanulus* genome annotation yielded 26,420 genes and 29,745 transcripts using the GALBA pipeline, with a single-copy BUSCO completeness of 75.28% and duplicated completeness of 22.32%, for a total completeness of 97.6% (Table 2). Similarly, in *C. caryae*, 31,436 genes and 34,679 transcripts were annotated, with 75.52% single-copy completeness, and 22.13% duplicated completeness, for a total completeness of 97.65% (Supplementary Table 2). For reference, the compiled reference annotations used in the GALBA pipeline included 160,472 proteins from 7 species with 99.86% total BUSCO completeness.

Blast2GO assigned Gene Ontology (GO) terms to 76% of predicted *C. nanulus* genes, with 53% receiving functional annotations. For *C. caryae,* 75% of genes were assigned GO terms, and 50% were functionally annotated.

### Genome comparison

The *C. nanulus* assembly ranked among the highest-quality of the 24 publicly available curculionid genomes. It was amongst the 4 highest contig N50 and 9 highest BUSCO completeness scores (Supplementary Table 3).

## Discussion

### Genomic resources for studying co-evolution

Species evolve within ecological networks shaped by complex webs of interspecies interactions. For seed-parasitic insects like *Curculio*, evolutionary trajectories are intimately tied to their host plants. The newly assembled genome of *C. nanulus* provides an essential resource for studying co-evolutionary processes at the genomic level. We have also observed that *C. nanulus* parasitizes at least 2 species of oaks: the mossycup oak *Quercus macrocarpa* and the English oak *Quercus robur*. This species’ generalist rather than host-specific strategy for parasitism is important context for future analyses regarding the role of host fidelity in diapause evolution.

*Curculio* species can profoundly influence forest biodiversity by reducing the reproductive success of dominant tree species, potentially benefiting understory or competing plants (Li et al. 2021). The ecological consequences of this predation depend on the host species targeted and the broader forest context. High-quality genome assemblies offer a means to investigate the genomic basis of plant-feeding habits in seed predators, and how the actions of such herbivores shape plant communities.

This assembly also enables investigation into the genomic basis of diapause, a key adaptation in *Curculio*. Diapause synchronizes weevil emergence with host seed availability, enabling persistence through masting cycles, with populational variation ensuring that at least some proportion of the population will emerge in a masting year. The length of diapause is controlled by both environmental and genetic factors. For example, in the chestnut weevil *Curculio elephas*, plasticity in diapause duration is greatly affected by the larval weight, fat stores, and emergence time, with larger and late-emerging larvae being prone longer diapause (Menu and Desouhant 2002). Additionally, *Curculio sikkimensis* experiences a lower probability of multi-year diapause when exposed to 5 °C for longer duration (Higaki and Toyama 2012).

However, the genetic mechanisms of influencing diapause length in *Curculio* remain unknown. Genes regulating circadian rhythms have been implicated in diapause control in arthropods, such as *Drosophila* (Yamada and Yamamoto 2011) and *Daphnia* (Schwarzenberger et al. 2020). Circadian clock genes such as these are likely candidates for similar diapause-regulating functions in *Curculio*. Understanding how these genes evolve in response to host phenology may reveal how quickly *Curculio* can shift hosts in response to environmental change or host decline. This, in turn, will improve predictions of co-extinction risk and resilience in host-parasite systems.

### Repetitive elements

Repetitive elements are a dominant feature of eukaryotic genomes and play a central role in genome evolution, regulation, and architecture (Bourque et al. 2018; Gilbert et al. 2021). For instance, repetitive elements have been found to influence 3-dimensional genomic architecture in ground beetles, as well as strongly reflect species boundaries (Sproul et al. 2020). Our analysis shows that repeat expansion is a defining feature of *Curculio* genome evolution. The substantial repeat content and its continuous expansion suggests that rapidly evolving repeat families contribute significantly to genome size differences within the genus. In particular, the expansion of unclassified elements is notably higher within *Curculio* than in other groups analyzed, indicating that the unclassified elements may play a unique role in *Curculio* evolution.

Repeat expansion in *Curculio* and its close relatives appears to have occurred independently of other curculionid lineages, indicating a lineage-specific dynamic. Notably, the large difference in genome size between *C. nanulus* and *C. caryae* can be attributed almost entirely to differences in repeat content, especially unclassified elements, LINEs, DNA transposons, and LTR retrotransposons. These genome assemblies and annotations are valuable resources for characterizing undescribed repeats in non-model insects and understanding how repeat dynamics influence genome evolution, species divergence, and adaptation in the species rich family Curculionidae.

## Supplementary Material

jkaf292_Supplementary_Data

## Data Availability

This Whole Genome Shotgun project for *Curculio nanulus* has been deposited at DDBJ/ENA/GenBank under the accession JBEWYK000000000. The version described in this paper is version JBEWYK010000000. The PacBio HiFi reads are available under BioProject PRJNA1129540. Annotations, intermediate assemblies, COI alignments, and tree files are available at https://doi.org/10.6084/m9.figshare.30546164 The PacBio HiFi reads generated by the Ag100Pest Initiative for *Curculio caryae* are available in sequencing run SRR18245025. Supplemental material available at G3 online.
