# Supplementary material for: Whole-genome assembly and annotation of the acorn weevil, Curculio nanulus (Coleoptera: Curculionidae)
Source: G3 (Bethesda). 2025 Dec 6;16(2):jkaf292. doi: 10.1093/g3journal/jkaf292 (PMC12869062; doi:10.1093/g3journal/jkaf292)
Supplement: jkaf292_Supplementary_Data [file jkaf292_supplementary_data.zip › Figure_S3_G3-2025-406368.pdf]

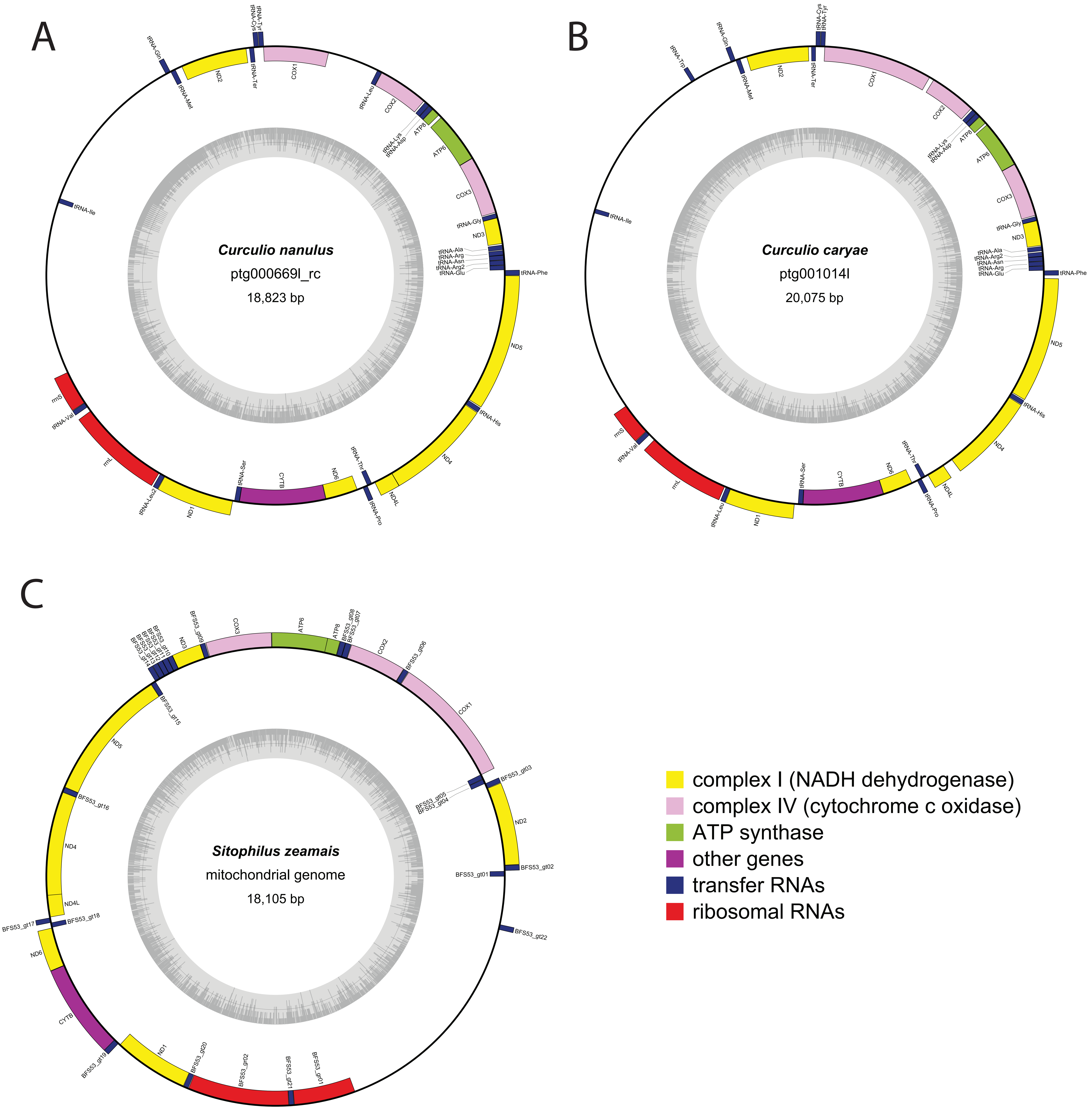
A
Curculio nanulus
ptg000669l_rc
18,823 bp
COX1
ND2
tRNA-Gln
tRNA-Met
tRNA-Tyr
tRNA-Cys
tRNA-Ter
tRNA-Leu
COX2
tRNA-Lys
tRNA-Asp
ATP8
ATP6
COX3
tRNA-Gly
ND3
tRNA-Ala
tRNA-Arg
tRNA-Asn
tRNA-Arg2
tRNA-Glu
tRNA-Phe
ND5
tRNA-His
ND4
ND4L
tRNA-Thr
tRNA-Pro
ND6
CYTB
tRNA-Ser
ND1
tRNA-Leu2
rrnL
tRNA-Val
rrnS
tRNA-Ile
B
Curculio caryae
ptg001014l
20,075 bp
tRNA-Cys
tRNA-Tyr
tRNA-Trp
tRNA-Gln
tRNA-Met
ND2
tRNA-Ter
COX1
COX2
tRNA-Lys
tRNA-Asp
ATP8
ATP6
COX3
tRNA-Gly
ND3
tRNA-Ala
tRNA-Arg2
tRNA-Asn
tRNA-Arg
tRNA-Glu
tRNA-Phe
ND5
tRNA-His
ND4
ND4L
tRNA-Thr
tRNA-Pro
ND6
CYTB
tRNA-Ser
ND1
tRNA-Leu
rrnL
tRNA-Val
rrnS
tRNA-Ile
C
Sitophilus zeamais
mitochondrial genome
18,105 bp
COX3
ATP6
ATP8
BFS53_gt08
BFS53_gt07
COX2
BFS53_gt06
COX1
BFS53_gt09
ND3
BFS53_gt10
BFS53_gt11
BFS53_gt12
BFS53_gt13
BFS53_gt14
BFS53_gt15
BFS53_gt03
BFS53_gt05
BFS53_gt04
ND2
BFS53_gt02
BFS53_gt01
BFS53_gt22
ND5
BFS53_gt16
ND4
ND4L
BFS53_gt17
BFS53_gt18
ND6
CYTB
BFS53_gt19
ND1
BFS53_gt20
BFS53_gr02
BFS53_gt21
BFS53_gr01
complex I (NADH dehydrogenase)
complex IV (cytochrome c oxidase)
ATP synthase
other genes
transfer RNAs
ribosomal RNAs
