# Supplementary material for: Whole-genome assembly and annotation of the acorn weevil, Curculio nanulus (Coleoptera: Curculionidae)
Source: G3 (Bethesda). 2025 Dec 6;16(2):jkaf292. doi: 10.1093/g3journal/jkaf292 (PMC12869062; doi:10.1093/g3journal/jkaf292)
Supplement: jkaf292_Supplementary_Data [file jkaf292_supplementary_data.zip › Supplemental_Material_G3-2025-406368.docx]

# Supplemental Material

## Results for *C. caryae* assembly

Publicly available PacBio HiFi reads for *Curculio caryae* totaled 92.7 Gbp across 10,074,085 reads. GenomeScope 2.0 predicted a genome length of ~1.6 Gbp, with ~62% repeat content and ~1% heterozygosity. The initial assembly (Table S2) consisted of 2.2 Gbp across 1,146 contigs, with 42x coverage, a contig L50 of 131, and a contig N50 of 5.02 Mbp. After refinement, the assembly length was reduced to 2.1 Gbp across 826 contigs, with slightly improved metrics: 43x coverage, contig L50 of 125, and contig N50 of 5.12 Mbp.

Completeness metrics for the *C. caryae* assembly remained high after refinement. While the total BUSCO completeness score decreased from 98.82% to 98.59%, complete and single-copy BUSCOs rose from 97.41% to 97.79%, and duplicated BUSCOs dropped from 1.41% to 0.80%. Fragmented and missing genes remained unchanged, indicating minimal compromise to completeness while removing redundancy and contaminants.

## Figures

**Figure S1** Photographs of *Curculio nanulus.* **A)** Lateral view of adult (specimen V0017). **B)** Lateral view of adult (specimen V0024).

**Figure S2** Phylogeny of *Curculio nanulus* specimens constructed using COI-5P barcodes placing the genome specimen (JBEWYK010000000) within *C. nanulus.* V0007 and V0008 are larval specimens collected from the same tree concurrent with JBEWYK010000000. V0015 and V0016 were collected from a nearby oak tree, reared to adulthood, and identified to the species level.

**Figure S3** Visualizations of the mitochondrial genome assemblies generated with OrganellarGenomeDraw v1.3.1. A) *Curculio nanulus*; B) *C. caryae*; C) *Sitophilus zeamais*, the reference mitochondrial genome.

##

## Tables

| **Table S1** Pairwise distances between the COI-5P barcodes of identified *C. nanulus* specimens and the COI gene extracted from the likely *C. nanulus* genome. | | | | | | |
| --- | --- | --- | --- | --- | --- | --- |
|  | **V0007** | **V0008** | **V0015** | **V0016** | **JBEWYK010000000** | **SRR18245025**  **(*Curculio caryae*)** |
| **V0007** | **-** |  |  |  |  |  |
| **V0008** | 0.0000000000 | - |  |  |  |  |
| **V0015** | 0.0000000000 | 0.0000000000 | - |  |  |  |
| **V0016** | 0.0016666667 | 0.0030395137 | 0.0018416206 | - |  |  |
| **JBEWYK010000000** | 0.0000000000 | 0.0000000000 | 0.0000000000 | 0.0030395137 | - |  |
| **SRR18245025**  **(*Curculio caryae*)** | 0.1150000000 | 0.1170212766 | 0.1123388582 | 0.1139817629 | 0.1190938511 | - |

| **Table S2** Contiguity and completeness statistics for the *Curculio caryae* initial, intermediate and final genome assemblies as well as completeness for gene annotations. Sequencing coverage was calculated by dividing the number of base pairs in the raw sequencing reads by the assembly size. Compleasm was run with the endopterygota_odb10 database. | | | |
| --- | --- | --- | --- |
|  | ***Curculio caryae*** | | |
| **Sequencing reads (bp)** | 92,686,342,389 | | |
| **Reads count** | 10,074,085 | | |
| **Mean read length** | 9,200.5 | | |
| **Read N50** | 10,381 | | |
|  | **Initial Assembly** | **Post-PurgeDups** | **Decontaminated with Blobtools** |
| **Contig count** | 1146 | 864 | 826 |
| **L50** | 131 | 128 | 125 |
| **N50** | 5,021,767 | 5,099,020 | 5,121,652 |
| **% GC content** | 35.4059 | 35.397 | 35.160 |
| **Shortest contig (bp)** | 5,207 | 5,207 | 5,339 |
| **Mean contig (bp)** | 1,927,898.5 | 2,521,315.3 | 2,596,665.7 |
| **Median contig (bp)** | 841,781 | 1,434,915 | 1,502,193 |
| **Longest contig (bp)** | 27,825,805 | 27,825,805 | 27,825,805 |
| **Total length (bp)** | 2,209,371,675 | 2,178,416,419 | 2,144,845,900 |
| **sequencing coverage** | 42x | 43x | 43x |
| **BUSCOs (compleasm)** |  |  |  |
| Complete (%) | 98.82 | 98.73 | 98.59 |
| Single copy (%) | 97.41 | 97.79 | 97.79 |
| Duplicated (%) | 1.41 | 0.94 | 0.80 |
| Fragmented (%) | 0.56 | 0.66 | 0.61 |
| Missing (%) | 0.61 | 0.61 | 0.80 |
| **Annotation** |  | | |
| Putative genes | 31,436 | | |
| Putative transcripts | 34,679 | | |
| Complete (%) | 97.65 | | |
| Single copy (%) | 75.52 | | |
| Duplicated (%) | 22.13 | | |
| Fragmented (%) | 0.56 | | |
| Missing (%) | 1.79 | | |

**Table S3** Assembly statistics for each of the 24 species of weevil (Curculionidae) genomes represented in GenBank, as well as the two *Curculio* genomes assembled in this study (rows bolded and highlighted). Rows are ordered by Contig N50 measured in kilobase pairs.

| **Assembly Accession** | **Organism Name** | **BUSCO**  **Complete** | **Coverage** | **Contig L50** | **Contig N50 (Kbp)** | **GC content** | **Contig count** | **Genome Size (Mbp)** |
| --- | --- | --- | --- | --- | --- | --- | --- | --- |
| GCA_030347505.1 | *Rhynchophorus ferrugineus* | 99.25% | 100 | 13 | 20,155.5 | 33.671 | 744 | 778.73 |
| GCA_935413205.1 | *Polydrusus cervinus* | 99.34% | 38 | 12 | 19,113.4 | 32.769 | 75 | 713.36 |
| GCA_030068095.1 | *Anthonomus grandis thurberiae* | 99.01% | 11 | 25 | 9,069.1 | 32.276 | 754 | 738.35 |
| **JBEWYK000000000** | ***Curculio nanulus*** | **98.90%** | **54** | **51** | **7,709.2** | **35.073** | **652** | **1,510.62** |
| GCA_022605725.3 | *Anthonomus grandis grandis* | 99.01% | 21 | 31 | 6,866.0 | 32.130 | 568 | 697.43 |
| GCA_016097725.1 | *Ips typographus* | 99.20% | 211 | 11 | 6,654.0 | 35.207 | 272 | 236.82 |
| **SRR18245025** | ***Curculio caryae*** | **98.59%** | **43** | **125** | **5,121.7** | **35.160** | **826** | **2,144.85** |
| GCA_917834065.1 | *Ceutorhynchus assimilis* | 98.21% | 40 | 51 | 3,538.5 | 35.294 | 628 | 674.66 |
| GCA_949748235.1 | *Platypus cylindrus* | 96.99% | 181 | 18 | 2,446.3 | 31.055 | 128 | 147.46 |
| GCA_963576705.1 | *Polydrusus tereticollis* | 99.01% | 15 | 274 | 1,537.9 | 34.042 | 1827 | 1,392.42 |
| GCA_958502075.1 | *Orchestes rusci* | 99.11% | 34 | 144 | 1,248.7 | 35.592 | 933 | 623.88 |
| GCA_951812265.1 | *Taphrorychus bicolor* | 99.20% | 31 | 128 | 1,227.0 | 36.233 | 1,283 | 575.09 |
| GCA_030620095.1 | *Kuschelorhynchus macadamiae* | 96.61% | 98 | 594 | 989.5 | 31.862 | 5,521 | 2,040.83 |
| GCA_024550625.1 | *Dendroctonus valens* | 96.47% | 806 | 82 | 985.5 | 36.833 | 1,143 | 320.96 |
| GCA_002938485.2 | *Sitophilus oryzae* | 98.73% | 101 | 257 | 723.2 | 32.632 | 5,424 | 757.90 |
| GCA_031761425.1 | *Cosmopolites sordidus* | 97.98% | 20 | 442 | 706.4 | 33.974 | 2,977 | 1,068.54 |
| GCA_019049505.1 | *Pachyrhynchus sulphureomaculatus* | 93.13% | 40 | 2,082 | 299.7 | 33.752 | 14,365 | 2,050.36 |
| GCA_018691245.2 | *Ips nitidus* | 96.23% | 94 | 285 | 175.9 | 35.173 | 33,250 | 231.34 |
| GCA_014170235.1 | *Listronotus bonariensis* | 94.35% | 38 | 2,726 | 120.3 | 31.308 | 22,298 | 1,112.42 |
| GCA_019359885.1 | *Listronotus oregonensis* | 87.62% | 67 | 6,281 | 53.1 | 30.955 | 44,498 | 1,292.90 |
| GCA_020466585.2 | *Dendroctonus ponderosae* | 97.93% | 747 | 2,136 | 26.1 | 35.803 | 20,279 | 213.86 |
| GCA_016904865.1 | *Pissodes strobi* | 80.79% | 70 | 21,576 | 24.9 | 35.390 | 144,278 | 1,831.48 |
| GCA_001012855.1 | *Hypothenemus hampei* | 97.70% | 100 | 18,102 | 1.9 | 32.255 | 219,563 | 130.40 |
| GCA_014849505.1 | *Elaeidobius kamerunicus* | 40.49% | 17 | 92,726 | 0.8 | 31.703 | 478,729 | 263.84 |
