# Supplementary figures and images for: Whole-genome assembly and annotation of the acorn weevil, Curculio nanulus (Coleoptera: Curculionidae)
Source: G3 (Bethesda). 2025 Dec 6;16(2):jkaf292. doi: 10.1093/g3journal/jkaf292 (PMC12869062; doi:10.1093/g3journal/jkaf292)

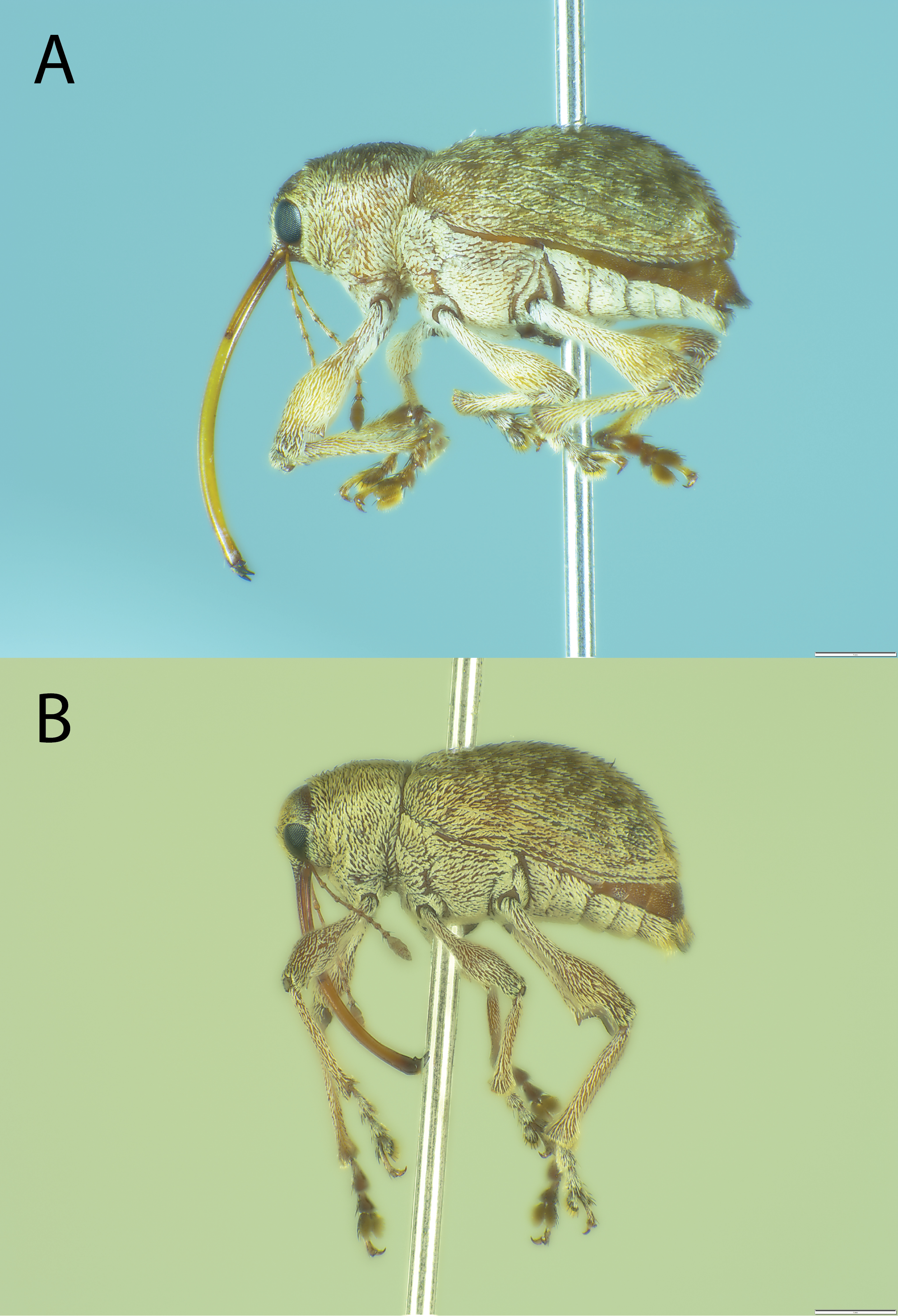

Supplement: jkaf292_Supplementary_Data [file jkaf292_supplementary_data.zip › Figure_S1_G3-2025-406368.png]

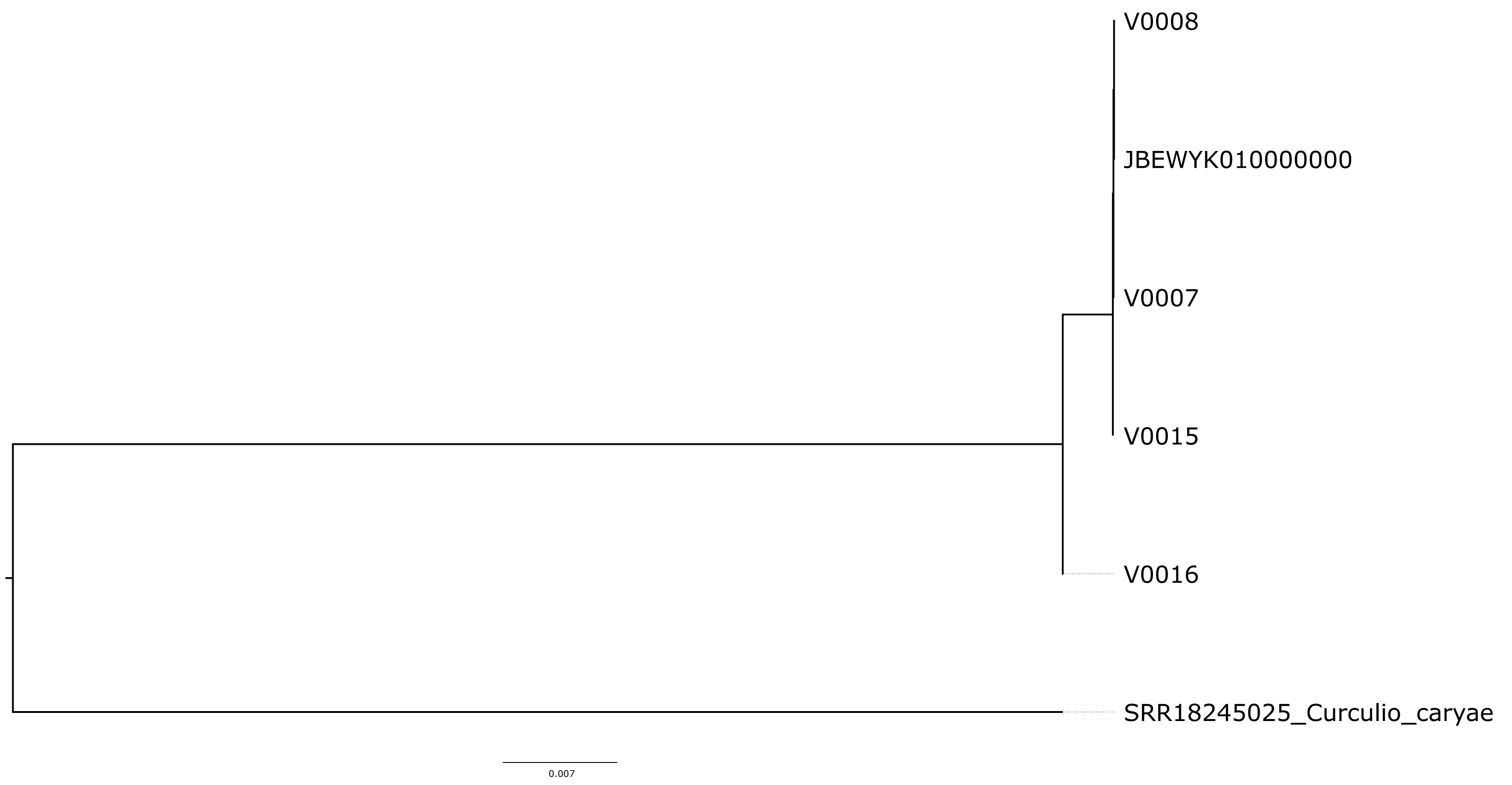

Supplement: jkaf292_Supplementary_Data [file jkaf292_supplementary_data.zip › Figure_S2_G3-2025-406368.pdf]
